# Glucagon-like Peptide-2 Depresses Ileal Contractility in Preparations from Mice through Opposite Modulatory Effects on Nitrergic and Cholinergic Neurotransmission

**DOI:** 10.3390/ijms25031855

**Published:** 2024-02-03

**Authors:** Eglantina Idrizaj, Cristina Biagioni, Chiara Traini, Maria Giuliana Vannucchi, Maria Caterina Baccari

**Affiliations:** 1 Section of Physiological Sciences, Department of Experimental & Clinical Medicine, University of Florence, 50139 Florence, Italy; mcaterina.baccari@unifi.it; 2 Research Unit of Histology & Embryology, Department of Experimental & Clinical Medicine, University of Florence, 50139 Florence, Italy; cristina.biagioni@unifi.it (C.B.); chiara.traini@unifi.it (C.T.)

**Keywords:** glucagon-like peptide-2 (GLP-2), cholinergic neurotransmission, nitric oxide, ileal contractile activity, neuromodulation

## Abstract

Glucagon-like peptide-2 (GLP-2) has been reported to influence gastrointestinal motor responses, exerting a modulatory role on enteric neurotransmission. To our knowledge, no data on GLP-2 effects on the motility of the isolated ileum are available; therefore, we investigated whether GLP-2 affects the contractile activity of mouse ileal preparations and the neurotransmitters engaged. Ileal preparations showed tetrodotoxin (TTX)- and atropine-insensitive spontaneous contractile activity, which was unaffected by the nitric oxide synthesis inhibitor, L-NNA. GLP-2 depressed the spontaneous contractility, an effect that was abolished by TTX or L-NNA and not influenced by atropine. Electrical field stimulation induced TTX- and atropine-sensitive contractile responses, which were reduced in amplitude by GLP-2 even in the presence of L-NNA. Immunohistochemical results showed a significant increase in nNOS-positive fibers in the ileal muscle wall and a significant decrease in ChAT-positive myenteric neurons in GLP-2-exposed preparations. The present results offer the first evidence that GLP-2 acts on ileal preparations. The hormone appears to depress ileal contractility through a dual opposite modulatory effect on inhibitory nitrergic and excitatory cholinergic neurotransmission. From a physiological point of view, it could be hypothesized that GLP-2 inhibitory actions on ileal contractility can increase transit time, facilitating nutrient absorption.

## 1. Introduction

Glucagon-like peptide-2 (GLP-2) is a 33-amino acid peptide generated from the prohormone proglucagon and, in addition to pancreatic alpha cells and the brain, is found to be secreted from the intestinal enteroendocrine L-type cells of ileal and colonic mucosa [1,2]. Several stimuli have been reported to modulate L-cell secretion in vivo and in vitro [3], including nutrients that represent a major stimulus for GLP-2 secretion into the bloodstream [4,5].

GLP-2 effects are mediated by its interaction with a specific receptor (GLP-2R), a G protein-coupled receptor expressed in both humans and animals at either the central or the peripheral level [1,6,7], including the gastrointestinal tract [8,9,10]. In the latter, GLP-2R expression occurs in enteroendocrine and subepithelial cells, myofibroblasts and in myenteric neurons [11]. GLP-2R has been shown to have a heterogeneous distribution in the gastrointestinal tract, with the highest levels of expression in the jejunum, followed by the duodenum, ileum, colon and stomach [9,12,13,14,15]. In agreement with this broad distribution of GLP-2R, the hormone has been reported to play a wide range of physiological roles in the gastrointestinal tract both in humans and animals, directed to a positive energy balance [16,17,18]. It performs enterotrophic actions on the small intestine mucosa, increasing the mesenteric blood flow, the secretion of various digestive enzymes and the uptake of nutrients [19,20,21,22,23,24,25,26]. Absorption processes are known to be closely related to gastrointestinal motor phenomena. In this view, GLP-2 has been observed to influence gastrointestinal motility by acting not only centrally but also peripherally [27,28]. In fact, in vitro studies demonstrated that the hormone exerts inhibitory effects through a direct neuromodulatory action on the enteric nervous system of mammals [29,30,31,32,33,34], in agreement with the localization of GLP-2R immunoreactivity in the cell bodies of myenteric neurons [11,31,32].

The inhibitory actions of GLP-2 at the stomach level [29,33,34] have been proposed by Amato and collaborators [29] to slow the flow through the pylorus, thus inducing delayed gastric emptying, which could, in turn, be an advantageous effect for improving intestinal nutrient absorption.

In addition to gastric emptying slowing, GLP-2 has been reported to increase intestinal transit time in mice [30]. Moreover, GLP-2 has also been demonstrated to depress duodenal contractility in isolated mouse preparations [31]. On the other hand, a depressed contractility of the other portions of the small intestine may also prolong the permanence of the luminal content in the upper proximal regions, thus favoring nutrient absorption.

Furthermore, immunohistochemical investigations looking for changes in neurotransmitter expression in the enteric neurons after GLP-2 exposition either in vivo or in vitro have shown that this hormone has a modulatory role on either the excitatory cholinergic or inhibitory nitrergic neurotransmitters in the stomach, duodenum and colon [29,31,33,34].

However, despite the observation that GLP-2 production occurs in the ileum in which GLP-2Rs are also highly expressed [9], to our knowledge, data on the effects of GLP-2 on the motility of this isolated intestinal segment are missing in the literature. Therefore, the present study aimed to investigate whether GLP-2 acts on ileal preparations to influence their contractility and whether the GLP-2 effects are due to changes in cholinergic and nitrergic neurotransmission. To this purpose, the effects of GLP-2 on either the spontaneous or neurally induced contractions of mouse ileal preparations were tested.

## 2. Results

### 2.1. Effects of GLP-2 on the Amplitude of Ileal Spontaneous Contractile Activity

**Figure 1:**
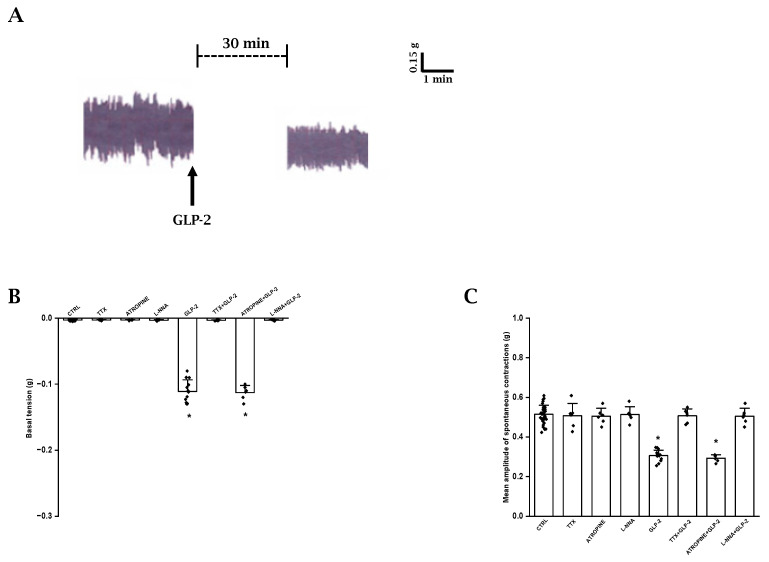
Effects of GLP-2 on basal tension and amplitude of spontaneous contractions in ileal preparations. (**A**): Typical tracing showing spontaneous contractile activity (left-hand trace). GLP-2 (20 nmol/L) causes a slight decay of the basal tension and reduces the amplitude of the spontaneous contractions (right-hand trace). (**B**,**C**): Influence of GLP-2 on basal tension (**B**) and mean amplitude of the spontaneous contractions (**C**). TTX, atropine and L-NNA have no effects on either parameter (**B**,**C**). GLP-2 elicits a slight decay of the basal tension (**B**) and a reduction in amplitude of the spontaneous contractions (**C**) compared to their own controls (CTRL). The inhibitory effects of the hormone are abolished in the presence of TTX (TTX + GLP-2) or L-NNA (L-NNA + GLP-2) and are unaffected by atropine (ATROPINE + GLP-2). Note the different values on y axes in the two histograms. Values recorded after the addition of each substance to the bath medium were compared with their own controls. Since no statistical differences were obtained in control values, for the two parameters, recorded data were analyzed together (CTRL). All values are the mean ± SE of 6–8 preparations. * *p* < 0.05 vs. the other groups of values and *p* > 0.05 between GLP-2 and atropine + GLP-2 (one-way ANOVA and Newman–Keuls post-test).

As previously observed [35], ileal preparations (*n* = 33) exhibited a spontaneous activity consisting of phasic rhythmic contractions (mean amplitude 0.51 ± 0.04 g) (Figure 1A), which were not influenced by TTX (1 μmol/L, *n* = 6) or atropine (1 µmol/L, *n* = 6) (mean amplitude 0.50 ± 0.06 and 0.50 ± 0.04 g, respectively; *p* > 0.05) (Figure 1C), indicating their myogenic and non-cholinergic nature, respectively.

In order to investigate the possible influence of a basal NO release on spontaneous contractile activity, the NO synthesis inhibitor L-NNA was added to the bath medium (*n* = 6). L-NNA (200 µmol/L) did not affect (*p* > 0.05) either the basal tension or the amplitude of the spontaneous contractions (Figure 1B,C), indicating the absence of a spontaneous NO release.

GLP-2 (20 nmol/L; *n* = 15) caused a slight decay of the basal tension (0.11 ± 0.01 g) and a reduction in the amplitude (mean amplitude 0.31 ± 0.03 g) of the spontaneous contractions compared to the controls (*p* < 0.05) (Figure 1A–C).

The depressant actions of GLP-2 (20 nmol/L) on spontaneous contractile activity were not affected by 1µmol/L atropine (*p* > 0.05; *n* = 6) (Figure 1B,C).

GLP-2 (20 nmol/L) added to the bath medium in the presence of 1 μmol/L TTX (*n* = 6) or 200 µmol/L L-NNA (*n* = 6) no longer affected the basal tension or the amplitude of the spontaneous contractions (Figure 1B,C), strongly suggesting that the hormone acts at the nervous level and that the nitrergic neurotransmission is involved in its depressant effects.

### 2.2. Influence of GLP-2 on the Amplitude of the Neurally Induced Contractile Responses Elicited by Electrical Field Stimulation (EFS)

**Figure 2:**
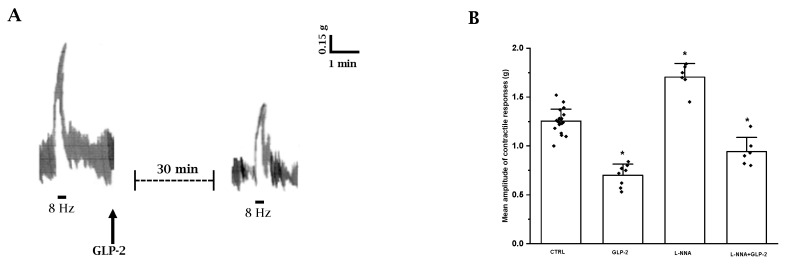
Influence of GLP-2 on the EFS-induced contractile responses in ileal preparations. (**A**): Typical tracing showing the contractile response to EFS (8 Hz) in mouse ileal preparations (left-hand trace). GLP-2 (20 nmol/L) reduces the amplitude of the neurally evoked contractile response (right-hand trace). (**B**): Influence of GLP-2 on the mean amplitude of the EFS-induced contractile responses in the absence or in the presence of L-NNA (200 µmol/L). GLP-2 (20 nmol/L) decreases the mean amplitude of the contractile responses to EFS compared to their own controls (CTRL). L-NNA increased the amplitude of the neurally induced contractile responses. In the presence of L-NNA(L-NNA + GLP-2), GLP-2 still reduces the amplitude of the contractile responses to EFS. Since no statistical differences were obtained in control values, data were analyzed together (CTRL). All values are mean ± S.E. of 6–8 preparations. * *p* < 0.05 vs. all the other groups of values (one-way ANOVA and Newman–Keuls post-test).

EFS elicited a fast phasic, rapidly declining contraction (mean amplitude 1.25 ± 0.15) superimposed to the spontaneous contractions (*n* = 20) (Figure 2A). The contractile response to EFS was abolished by 1 µmol/L TTX (*n* = 3) or 1 µmol/L atropine (*n* = 3), indicating its nervous and cholinergic nature, respectively. 

The amplitude of the EFS-induced contractile responses was greatly reduced (mean amplitude 0.72 ± 0.12; *p* < 0.05) by 20 nmol/L GLP-2 (*n* = 8) compared to controls (Figure 2A,B).

Based on the observation that nitrergic neurotransmission appears to be involved in the depressant effects of GLP-2 on spontaneous contractility, the influence of the hormone on the neurally induced contractile responses in the presence of L-NNA was also tested.

L-NNA (200 µmol/L) increased the amplitude of the contractile response to EFS (*n* = 6; *p* < 0.05) (Figure 2B), indicating the removal of an inhibitory nervous nitrergic control. GLP-2 (20 nmol/L) added to the bath medium 20 min following L-NNA (*n* = 6) still reduced the amplitude of the neurally induced contractile responses (Figure 2B), also indicating an inhibitory action of the hormone on excitatory cholinergic neurotransmission.

### 2.3. Influence of GLP-2 on Neuronal Nitric Oxide Synthase (nNOS) Expression in the Muscle Wall of Mouse Ileum

**Figure 3:**
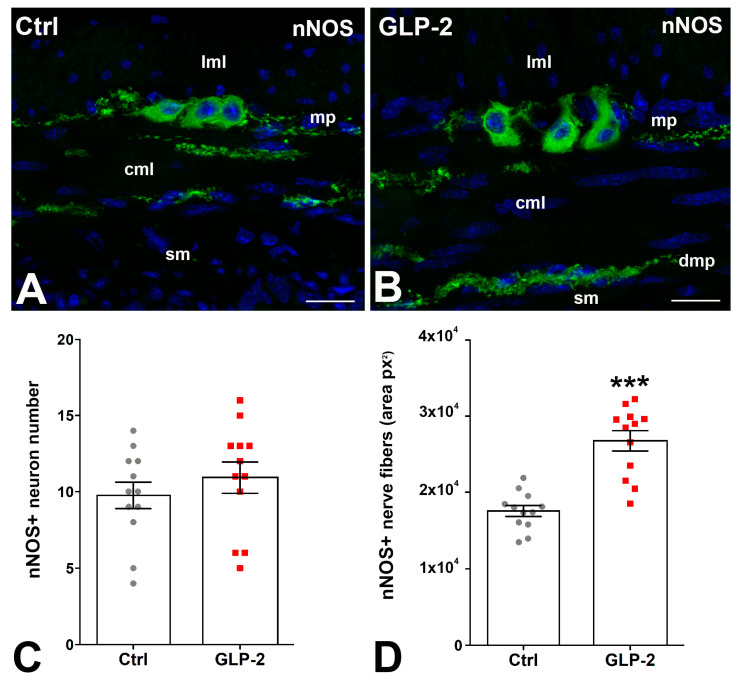
GLP-2 increases the number of nNOS nerve fibers in the ileal muscle wall. (**A**,**B**): Neuronal Nitric Oxide Synthase (nNOS) immunoreactivity (IR) in the muscle wall of ileum. In controls (**A**) and GLP-2-exposed (**B**) specimens, the labeling was located in the myenteric neurons and in the nerve fibers both at the myenteric plexus (mp) and in the circular muscle layer (cml). Bar = 20 µm. lml: longitudinal muscle layer; sm: submucosa; dmp: deep muscular plexus. (**C**,**D**): Quantitation of the nNOS-IR neuron number (**C**) showed no significant difference between the two groups, whereas a significant increase (*** *p* < 0.01; unpaired *t*-test) in nNOS-positive fibers (**D**) was detected in the GLP-2-exposed specimens; each dot represents the number of neurons in a section (**C**) or the px2 per section (**D**).

In control and GLP-2-exposed specimens, nNOS immunoreactivity (IR) was detected in the soma of some myenteric neurons and in numerous nerve fibers located along the myenteric nerve strands and in the circular muscle layer where they concentrated in correspondence with the deep muscular plexus (Figure 3A,B). Quantification of the labeling showed no difference in the number of nNOS-IR myenteric neurons between the two groups (Figure 3C); on the contrary, a significant increase in positive nerve fibers was seen in GLP-2-exposed specimens (Figure 3D).

### 2.4. Influence of GLP-2 on Choline Acetyl Transferase (ChAT) Expression in the Muscle Wall of Mouse Ileum

**Figure 4:**
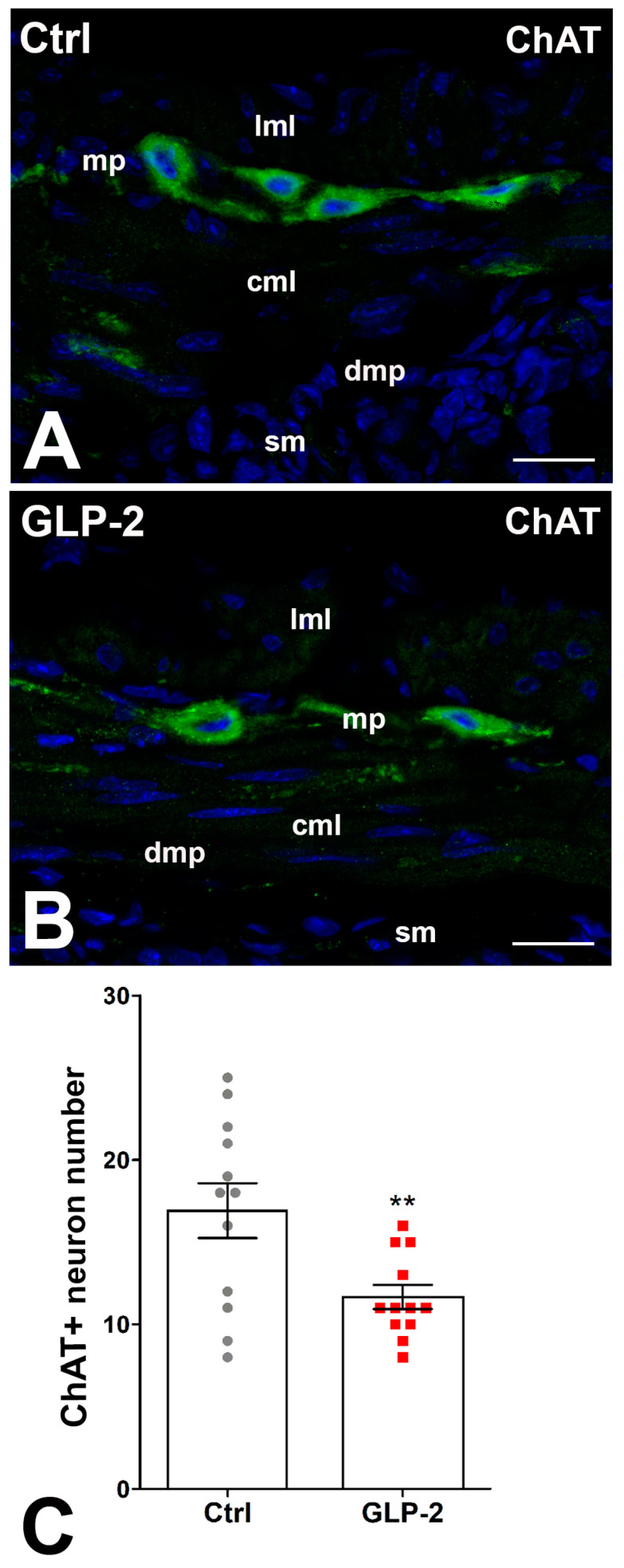
GLP-2 decreases the number of cholinergic neurons in the ileal myenteric plexus. (**A**,**B**): Choline Acetyl Transferase (ChAT) immunoreactivity (IR) in the muscle wall of ileum. In controls (**A**) and GLP-2-exposed (**B**) specimens, the labeling was mainly located in the myenteric neurons, while very few nerve fibers were labeled at the myenteric plexus (mp) and circular muscle layer (cml). Bar = 20 µm. lml: longitudinal muscle layer; sm: submucosa; dmp: deep muscular plexus. (**C**): Quantitation of the ChAT neuron number (**C**) showed a significant decrease in GLP-2-exposed specimens compared to controls (** *p* < 0.02; unpaired *t*-test); each dot represents the number of neurons in a section.

In control and GLP-2-exposed specimens, ChAT immunoreactivity (IR) was detected in numerous myenteric neurons, while few fibers showed IR. The labeling had a granular aspect in both sites (Figure 4A,B). The count of ChAT-positive neurons showed a significant decrease in the GLP-2-exposed specimens compared with the control ones (Figure 4C).

## 3. Discussion

The present results offer the first evidence that GLP-2 acts on the mouse ileum. In particular, the hormone appears to depress ileal contractility through a dual opposite modulatory effect on inhibitory nitrergic and excitatory cholinergic neurotransmission.

The observation that TTX, atropine or L-NNA did not affect the spontaneous activity of ileal preparations, other than indicating its muscular origin, suggested that a basal release of acetylcholine or NO from enteric nerves did not occur. On the other hand, GLP-2 caused a decay of the basal tension and a reduction in the amplitude of the spontaneous contractions that was abolished by TTX or L-NNA, indicating that its effects likely occur through a positive neuromodulatory action on nitrergic transmission.

GLP-2 also decreased the amplitude of the EFS-evoked contractile responses, which were “per se” abolished either by TTX or atropine, suggesting their nervous and cholinergic nature, respectively. Moreover, the increased amplitude of the neurally induced contractile responses to EFS caused by L-NNA indicates the removal of an inhibitory nitrergic component in agreement with reports showing that, although contractile responses are obtained during EFS, inhibitory nerve fibers are simultaneously activated with the excitatory ones [36,37,38,39].

Therefore, the reduction in amplitude of the contractile responses to EFS induced by GLP-2 could be ascribable, other than to a major activation of the inhibitory component, to a lower activation of the excitatory one. In this view, the observation that GLP-2 in the presence of L-NNA (i.e., when the nitrergic inhibitory component is ruled out) still depressed neurally induced cholinergic contractile responses, also indicating an inhibitory modulatory role of the hormone on cholinergic excitatory neurotransmission.

This action likely occurs through a reduction in acetylcholine release, which would explain the observation that ileal spontaneous activity and its depression by GLP-2 were unaffected by atropine since spontaneous acetylcholine release does not appear to occur in unstimulated preparations.

The mechanical recordings in ileal preparations exposed to GLP-2 clearly indicated an involvement of neurotransmission as confirmed by the morphological results. In particular, the increase in nNOS expression in the nerve fibers located in the circular muscle layer caused by GLP-2 can explain the abolition of the decay of the basal tension and the reduction in the amplitude of the spontaneous contractions caused by GLP-2 in the presence of TTX or L-NNA, whereas the decrease in the number of ChAT-positive myenteric neurons detected in the GLP-2-exposed preparations fits well with the hormone-induced reduction in the amplitude of EFS contractile responses even in the presence of L-NNA.

Many hormones in the gut have been reported to control the production of NO [40,41,42,43,44,45], which is considered one of the main inhibitory neurotransmitters released from non-adrenergic, non-cholinergic nerve fibers and causes smooth muscle relaxation [36,37,38,46,47].

In experimental conditions comparable to the present ones, we demonstrated that GLP-2 increased the number of nNOS neurons and fibers in the muscle wall of mouse gastric fundus [33]; furthermore, chronic treatment with GLP-2 of mice subjected to chemotherapy protected the nitrergic neurons of the myenteric plexus from damage, suggesting an elective trophic action of the hormone toward these neuronal cells [48].

Indeed, the presence of GLP-2R has been reported both on nitrergic and cholinergic enteric neurons of the mouse small intestine and colon [9,27,31,32]. The present data suggest that in the ileum, both ChAT and nNOS neurons might express GLP-2R. If so, it has to be inferred that the cellular mechanisms activated by the hormone on these two types of neuronal populations are opposite. In this view, some studies have shown that GLP-2R can couple, alternatively, to a G protein that increases intracellular cAMP, facilitating neurotransmission, or to a G protein responsible for a decrease in cAMP concentration and neurotransmitter inhibition [49,50]. Moreover, the demonstration that the inhibitory effects of GLP-2 on evoked cholinergic contractions in the mouse colon were significantly repressed by pertussis toxin, a Gi/o protein inhibitor, supported the hypothesis that the GLP-2R may be coupled to an inhibitory G protein [32]. In summary, we suppose that in our ileum preparations, the GLP-2R on the nNOS neurons binds to an excitatory G protein, whereas on the ChAT neurons, it recruits an inhibitory G protein.

Although the present experiments indicate that GLP-2 depresses ileal contractility by exerting a modulatory role on both cholinergic and nitrergic neurotransmission, the possible involvement of an increased release also of other inhibitory neurotransmitters cannot be completely excluded. In this view, GLP-2 has been reported to cause gastrointestinal relaxation by releasing vasoactive intestinal peptides from enteric motor neurons [29,34], which, in turn, is also able to induce muscular NO production [51]. This further mechanism could in any case agree with the abolition of the depressant effects of GLP-2 on ileal spontaneous contractility by L-NNA observed in the present experiments.

In conclusion, the results of the present experiments offer the first evidence that GLP-2 acts on ileal preparations and depresses their contractility as a result of complex interactions with inhibitory nitrergic and excitatory cholinergic pathways. From a physiological point of view, it could be hypothesized that the inhibitory action of GLP-2 on ileal contractility increases ileal transit time, thus prolonging the permanence of the luminal content in the more proximal intestinal regions to favor nutrient absorption. This function is in accordance with the reported role of the hormone in the small intestine [4,16,19,22] and the successful introduction of a GLP-2 analog in the treatment of patients affected by short bowel syndrome [3,15,52]. Moreover, the inhibitory effects of GLP-2 on ileal contractility could, at the same time, lead to an increased contact time of nutrients with enteroendocrine cells to increase hormone release, as occurs in the ileal brake reflex [53,54], also generating a positive feedback loop of its own release.

## 4. Materials and Methods

### 4.1. Animals and Ethical Approval

Experiments were performed on 8- to 12-week-old female mice (C57BL/6J; ENVIGO, Udine, Italy). The mice were fed with standard laboratory chow and water and were housed under a 12 h light/12 h dark photoperiod and controlled temperature (21 ± 1 °C). The experimental protocol was designed in compliance with the guidelines of the European Communities Council Directive 2010/63/UE and the recommendations for the care and use of laboratory animals approved by the Animal Care Committee of the University of Florence, Italy, with authorization from the Italian Ministry of Health (code 0DD9B.N.ZB6/2020). The mice were killed by prompt cervical dislocation to minimize animal suffering. As previously reported [35], the abdomen was immediately opened, the distal ileum was removed and its luminal content was gently flushed out with a physiological solution.

### 4.2. Functional Studies

Segments of the distal ileum (within 30 mm from the ileocecal valve) were isolated. Whole full-thickness ileal segments (10 mm in length), transversely cut, were dissected and mounted in 5 mL double-jacketed organ baths containing Krebs–Henseleit solution, which consisted of 118 mM NaCl, 4.7 mM KCl, 1.2 mM MgSO_4_, 1.2 mM KH2PO_4_, 25 mM NaHCO_3_, 2.5 mM CaCl_2_ and 10 mM glucose (pH 7.4), and bubbled with 95% O_2_/5% CO_2_. Prewarmed water (37 °C) was circulated through the outer jacket of the tissue bath via a constant-temperature circulator pump. The temperature of the Krebs–Henseleit solution in the organ bath was maintained within ±0.5 °C.

The preparations were mounted in the direction of the longitudinal muscle layer: one end of each preparation was tied to a platinum rod, while the other was connected to a force-displacement transducer (Grass model FT03, Quincy, MA, USA) by a silk thread for continuous recording of isometric tension. The transducer was coupled to polygraph systems (Grass model 7K, Quincy, MA, USA). As previously reported [35], the preparations were allowed to equilibrate for at least 1 h under an initial load of 1.5 g. During this period, the preparations underwent repeated and prolonged washes with Krebs–Henseleit solution to avoid accumulation of metabolites in the organ baths.

Electrical field stimulation (EFS) was applied via two platinum wire rings (2 mm diameter, 5 mm apart) through which the preparation was threaded. Electrical pulses (rectangular waves, 80 V, 8 Hz, 0.5 ms, for 15 s) were provided by a Grass model S8 stimulator.

#### 4.2.1. Drugs

The following drugs were used: atropine (1 µmol/L), glucagon-like peptide-2 (GLP-2, 20 nmol/L), tetrodotoxin (TTX, 1 µmol/L) and L-NG-nitro arginine (L-NNA, 200 µmol/L). All drugs were obtained from Sigma Chemical (St. Louis, MO, USA) except for GLP-2, which was from Tocris Bioscience (Bristol, UK).

Solutions were prepared on the day of the experiment, except for TTX, for which stock solutions were kept stored at −20 °C.

Drug concentrations are referred to as final bath concentrations and are in the range of those previously reported to be effective in gastrointestinal preparations [33,34].

#### 4.2.2. Experimental Protocol

The first series of data was obtained by recording the spontaneous contractile activity of ileal preparations after the inclusion in the bath medium of the following drugs: the nerve-ending blocker tetrodotoxin (TTX), the muscarinic receptor antagonist atropine and the nitric oxide synthesis inhibitor NG-nitro-L-arginine (L-NNA).

The second series of data was obtained by evaluating the effects on the spontaneous motility of the addition of GLP-2 (20 nmol/L) to the bath medium. Since we previously observed [34] that, in strips from the mouse gastric fundus, the response of a subsequent addition of GLP-2 at 20 nmol/L to the bath medium was regained only after washing the preparation for 15 min, the same procedure was followed in the present experiments. The hormone was given alone or in the presence of the following drugs: TTX, atropine or L-NNA.

The third series of data was obtained by recording the response to EFS in the presence of TTX, atropine or L-NNA. The effects of the addition of GLP-2 to the bath medium on the neurally induced contractile responses were evaluated alone or in the presence of L-NNA.

#### 4.2.3. Data Analysis and Statistical Tests

The amplitude of either spontaneous contractions or neurally induced contractile responses is expressed as absolute values (grams) and was measured when the maximal effect was reached. Basal tension was evaluated as changes in the recording baseline and expressed as absolute values (grams). Values recorded after the addition of each substance to the bath medium were compared with their own controls. Since no statistical differences were obtained in control values, these data were analyzed together.

The statistical significance was assessed by one-way ANOVA followed by the Newman–Keuls post-test for multiple comparisons. A *p*-value < 0.05 was considered significant. Results are means ± SE. In the results, n indicates the number of preparations.

### 4.3. Morphological Studies

Distal ileum samples were taken and mounted in 5 mL organ baths containing Krebs–Henseleit solution. One half of the specimens was exposed for 30 min to GLP-2 (20 nmol/L). The second half was maintained in Krebs solution for the same period of time without the addition of the hormone (controls).

#### 4.3.1. Tissue Sampling

Ileum samples were immediately transferred to a fixative solution (10% formaldehyde solution (HT501320, Sigma-Aldrich, Merck, Darmstadt, Germany) overnight at 4 °C. The day after, the specimens were dehydrated in graded ethanol series, cleared in xylene and embedded in paraffin in order to cut transversal sections of the samples. Sections (thickness of 5 µm) were cut using a rotary microtome (HistoCore MULTICUT, Leica, Buccinasco, MI, Italy) and collected on positively charged slides.

#### 4.3.2. Immunohistochemistry

The sections, once deparaffinized in xylene and re-hydrated in descending ethanol series to distilled water, were treated for antigen retrieval for 20 min at 90–92 °C in Tris buffer (10 mM) with EDTA (1 mM, pH 9.0), followed by cooling at room temperature (RT). The sections were then washed in 0.1 M phosphate-buffered saline (PBS, pH 7.4) and blocked for 20 min with 5% normal donkey serum (NDS, Jackson Laboratories, Inc., Bar Harbor, ME, USA) in PBS. The sections were incubated in 5% NDS PBS ON at 4 °C in the presence of ChAT antibodies (goat polyclonal 1:200, Millipore Corporation, Temecula CA, USA) or nNOS antibodies (rabbit polyclonal 1:1000, Millipore Corporation). The next day, the sections were incubated for 2 h at RT in the dark with appropriate fluorochrome-conjugated secondary antibodies (donkey anti-goat AlexaFlour 488 Thermo Fisher Scientific, Rockford, IL, USA; or goat anti-rabbit AlexaFlour 488, West Grove, PA, USA) diluted 1:333 in 5% NDS PBS. Afterward, the sections were washed in PBS, incubated for 10 min with Hoechst 33342, a nuclei marker (20 μg/mL; B2261, Sigma-Aldrich, Merck, Darmstadt, Germany), dissolved in PBS, thoroughly washed in distilled water and mounted in an aqueous medium (FluoreGuard Mounting Medium, ScyTek Laboratories Inc., Logan, UT, USA). To exclude the presence of non-specific immunofluorescence labeling, negative controls were performed by omitting the primary antibody. Immune reactions were observed under an epifluorescence Olympus BX63 microscope (Olympus, Italy) or a Leica Stellaris 5 confocal microscope (Leica Microsystems, Mannheim, Germany) using 488 and 370 nm excitation wavelengths for the green and blue fluorescent labels, and the fluorescence images were captured using the corresponding digital camera (Olympys XM10) and image acquisition software (cellSens Dimension V0118 Olympus and LAS X Leica).

#### 4.3.3. Quantitation and Statistical Analysis

The counting of ChAT and nNOS immunoreactive (IR) neurons was performed by two observers, blind to each other, in the myenteric neurons, along the entire section (4 sections/animal; 3 animals/group). Only IR neurons expressing the nucleus marker (Hoechst 33342) were included. The results were expressed as positive neurons for section ± S.E.M. nNOS-IR fibers were quantified at the level of the myenteric plexus, excluding the nNOS-IR neuronal bodies, and in the circular layer. The images were acquired using a 20× objective, and the reconstruction of the entire section was performed using appropriate software. Using ImageJ software v1.53k (NIH, Bethesda, ML, USA), an ROI (region of interest) line corresponding to the perimeter of the circular muscle layer was drawn; the entire section was turned to 8-bit greyscale and the nNOS-IR fibers were identified after setting up the threshold; lastly, the corresponding pixels2 were calculated using the analyse tool of ImageJ v1.53k. The result was expressed as pixels2 for the section ± S.E.M. Statistical analysis was performed by an unpaired Student’s *t*-test. *p* < 0.05 was considered significant.

## Data Availability

Data are contained within the article.

## Abbreviations

cAMP	cyclic adenosine monophosphate
ChAT	choline acetyl transferase
CTRL	control
EFS	electrical field stimulation
GLP-2	glucagon-like peptide-2
GLP-2R	glucagon-like peptide-2 receptor
L-NNA L-NG	nitro arginine
NO	nitric oxide
nNOS	neuronal nitric oxide synthase
TTX	tetrodotoxin
